# Irisin Has a Protective Role against Osteoporosis in Ovariectomized Rats

**DOI:** 10.1155/2021/5570229

**Published:** 2021-04-26

**Authors:** Enas N. Morgan, Ashwag Saleh Alsharidah, Ayman M. Mousa, Husam M. Edrees

**Affiliations:** ^1^Department of Medical Physiology, College of Medicine, Zagazig University, Al-Sharquia 44519, Egypt; ^2^Department of Physiology, College of Medicine, Qassim University, Buraidah 52645, Saudi Arabia; ^3^Department of Basic Health Sciences, College of Applied Medical Sciences, Qassim University, Buraydah 52645, Saudi Arabia; ^4^Department of Histology and Cell Biology, Faculty of Medicine, Benha University, Benha 13518, Egypt; ^5^Department of Public Health, College of Public Health and Health Informatics, Qassim University, Al Bukairiyah 51264, Saudi Arabia

## Abstract

The reduction in estrogen levels results in a decrease in bone density at menopause. Irisin is a myokine that modulates the benefits of exercise, which may include bone health. This study was planned to examine irisin's impact in preventing osteoporosis after ovariectomy. 4 groups of female albino rats (10 rats/group): control, sham-operated, ovariectomized (OVX-control), and OVX-irisin-treated. Serum levels of bone markers [osteocalcin (OC), bone alkaline phosphatase (BALP), tartrate-resistant acid phosphatase (TRAP), calcium (Ca^++^), phosphorus (P)], glucose, and insulin were being measured. Body mass index, Homeostatic Model Assessment of Insulin Resistance (HOMA-IR), dry and ash femur weight, and bone contents of Ca^++^ and P were investigated. The femur was examined histopathologically. The OVX-control group showed an increase in serum levels of OC, BALP, TRAP, calcium, phosphorus, BMI, glucose, insulin, and HOMA-IR (*P* < 0.05) and a reduction in dry and ash weight of the femur, the concentration of calcium and phosphorus content in bone ash (*P* < 0.05). The OVX-irisin-treated group exhibited a decrease in serum levels of OC, BALP and TRAP, calcium, phosphorus, BMI, glucose, insulin, HOMA-IR (*P* < 0.05), and a rise in dry and ash weight of the femur, the concentration of calcium and phosphorus in bone ash (*P* < 0.05). Histological examination of the distal femur diaphysis of the OVX-irisin-treated group exhibited proper bone architecture and density compared with that of the OVX-control group. It is concluded that irisin treatment in the OVX rats safeguarded the regular bone architecture and normal levels of serum bone biomarkers. Irisin may be a possible novel target in the prohibition of postmenopausal osteoporosis.

## 1. Introduction

Physical activity is the main element in improving bone quality; many studies have declared that running and walking activities abate the process of aging and associated disorders like osteoporosis, diabetes, and obesity [1]. Recently, there is growing evidence that control of muscle over bone health is not only through the mechanical effect but also via chemical messengers termed myokines [2]. Irisin is one of these known myokine families that activates thermogenesis, rising energy expenditure, and bettering glucose homeostasis [3]. The main organ targeted for irisin is bone rather than white adipose tissue (WAT). An in vivo study determined that the injection of irisin weekly in mice increased the cortical bone mass and bone mechanical features by a dose 35 times lesser than that requested for WAT's browning [4].

Osteoblasts are responsible for the manufacture of bone matrix. They are mesenchymal in origin, have plentiful endoplasmic reticulum, and produce abundant alkaline phosphatase. The bone matrix is composed of organic (∼20% of the wet weight of bone) and inorganic components. Bone modeling and remodeling is a process that occurs throughout life through resorption by osteoclasts and construction by osteoblasts [5].

Osteoporosis is a skeletal illness with reduced bone density and bone quality, leading to a rise in bone fragility and fractures [6, 7] and deteriorating mobility and quality of life [8]. Osteoporosis occurs silently and progressively [9]. The prevalence of osteoporosis among Caucasian women aged above 50 years was between 7.9 and 22.6% [10]. A study that quantified the worldwide disability that came from osteoporotic fractures reported about 9.0 million fractures due to osteoporosis; the most significant number of osteoporotic fractures was in Europe (34.8%) [11].

Postmenopausal osteoporosis may be due to multiple factors, including the estrogen deficiency that disrupts the modeling and remodeling cycle through elevating resorption by osteoclasts with no consistent increase in bone formation, leading to bone loss [12, 13]. The estrogen deficiency leads to cellular changes in bone and augmented secretion of tumor necrosis factor (TNF*α*) with increased sensitivity to interleukin-1 (IL-1). TNF*α* and IL-1 stimulate stromal cells/preosteoblasts to secrete many cytokines that combine with receptor activators of nuclear factor B ligand (RANK ligand) expressed by osteoclasts and osteoclast precursors to promote osteoclast differentiation and represent the final common pathway for bone resorption [14]. In addition to age-related changes that start with the first phase of menopause causing negative calcium balance and develops in the sixties, leading to reduced calcium absorption and more negative calcium balance. The decrease in calcium absorption results in an elevation of bone resorption due to secondary hyperparathyroidism. With increasing age, calcium absorption becomes more difficult due to increased intestinal resistance to endogenous circulating 1, 25 dihydroxy vitamin D [15].

The association between menopause and metabolic syndrome (MS) was defined by Park et al. [16] who revealed that the risk of metabolic syndrome increased by 60% after menopause, even after adjusting different variables, like age, body mass index (BMI), and physical inactivity. The relation between metabolic syndrome (MS) and osteoporosis conditions is still inconsistent and contradictory. However, insulin resistance, a common feature of MS, may be considered a link between MS and chronic degenerative diseases, including osteoporosis [17].

Irisin is a peptide myokine released as a result of exercise [18, 19]. It is produced by the cleavage of type I membrane proteins and encoded by the fibronectin type III domain. This domain contains five genes called FNDC5, which consist of a signaling peptide (29-amino acids), a domain (94-amino acids), and a C-terminal (lysis performing site). In mice and humans, irisin has a 100% identity. Irisin was revealed by gas chromatography/mass spectrometry (GC/MS) analysis in human systemic circulation [20]. Tissues secreting irisin include skeletal muscle, adipose tissue, and cardiac muscle. Moreover, irisin is immunoreactive in ovaries, testes, salivary glands, stomach, neuronal cells, and sweat glands [21].

The principal function of irisin is thermogenesis regulation that is moderated by enhancing the receptors for peroxisome *ɣ* and its coactivator-1*α*, to trigger mitochondrial biogenesis [22]. Irisin increases energy expenditure, endorses loss of weight, and decreases diet-induced insulin resistance [23].

The impact of irisin on bone health was reported by Palermo et al. [24]. They showed that vertebral fractures due to postmenopausal osteoporosis are inversely correlated with irisin, irrespective of muscle and fat mass, bone mineral density, and physical activity. Colaianni et al. [25] reported that the administration of small doses of recombinant irisin to young male mice causes a reduction in osteoclasts and a surge in osteoblast genes' expression with diminution in the interpretation of its gene suppressors. That increases the anabolic activity in the bone mass and the mineral density of the cortical bone. They also reported a development in bone geometry through a rise in the periosteal perimeter. Moreover, irisin improves insulin resistance [26, 27] which may be a hidden cause of postmenopausal osteoporosis [17].

This study evaluated irisin injection's perspective effect on protecting against osteoporosis in ovariectomized rats and investigated its impact on bone architecture and biochemical markers.

## 2. Materials and Methods

### 2.1. Ethics Declaration

All steps of the experiments were endorsed by the Institutional Animal Care and Use Committee of Zagazig University (approval no: ZU-IACUC/7/F/4/2019), and all experiments were as stated in the guidelines authenticated in the Guide for the Care and Use of Laboratory Animals.

### 2.2. Tested Drug

Irisin (Fibronectin Type III Domain-containing Protein 5, Fibronectin Type III repeat-containing Protein 2, FNDC5) was bought from MyBiosource, Inc., sunny Southern California, San Diego (USA).

### 2.3. Animal and Experimental Design

Forty female albino rats (220 ± 30 g). The rats were housed and preserved at 21–23°C with free access to water at the Animal House, College of Medicine, Zagazig University, Egypt. Animals were fed standard rat chow. The acclimatization of rats to laboratory conditions was permitted for two weeks before beginning the study. The rats were weighed; then, the calculation of BMI was done according to this equation [bodyweight in gram/length from nose to anus in cm^2^] [28]. The rats were grouped equally into four groups (10 rats/group). All experimental rats in all groups were injected intramuscularly (i.m.) in the front of the thigh.

Group I (control): rats were injected with sterile injectable water (vehicle) for two weeks after the beginning of the experiment.

Group II (sham-operated): rats were sham-operated; two weeks later, they were injected with sterile injectable water for four weeks.

Group III (OVX-control): rats underwent bilateral ovariectomy to induce osteoporosis; two weeks later, they were injected with sterile injectable water (vehicle) for 4 weeks.

Group IV (OVX irisin-treated): rats were bilaterally ovariectomized as in group III; two weeks later, they were injected with irisin hormone (100 *μ*g/kg/week) for four weeks Colaianni et al. [25].

After completion of the experiment (6 weeks), rats were weighed and the final body mass index (BMI) was calculated from the following equation: BMI = body weight (g)/length^2^ (cm^2^) (from nose to anus) [28].

At the end of the experiments, rats were anaesthetized by thiopental sodium (15 mg/kg BW), blood samples were assembled from the rat tail vein and were left for 30 minutes to clot at room temperature and, then, centrifuged at 3000 rpm for 15 minutes, and the supernatant serum was buffered at -20°C until assay. After finishing blood sampling, all experimental animals were sacrificed by cervical dislocation; both femurs were dissected from each rat to be prepared for histopathological study.

### 2.4. Ovariectomy

Postmenopausal osteoporosis in rats was induced with bilateral ovariectomy [29]. Most researchers exhibited a significant bone loss in the proximal tibial metaphysis 14 days after ovariectomy [30, 31].

Animals of groups II, III, and IV were anesthetized using thiopental sodium (injected in a dose of 15 mg/kg BW). The lower abdomen was incised after being shaved. Fallopian tubes were tied below the ovaries with absorbable catgut sutures followed by removal of the ovaries. In the sham-operated group, a surgical incision was done to expose the ovaries by replacing them in the same position. The rats underwent postoperative care including the systemic administration of analgesics and antibiotics [32].

### 2.5. Biochemical Analysis

Serum glucose levels were estimated using glucose enzymatic liquizyme rat kits (Biotechnology, Egypt) according to Tietz [33], and insulin levels were estimated according to Temple et al. [34] using KAP1251- INS-EASIA rat Kits (BioSource Europe SA, Belgium). HOMA-IR (Homeostatic Model Assessment of Insulin Resistance Index) was calculated depending on serum insulin level (mIU/mL) and serum glucose level (mg/dL) by using the formula described by Matthews et al. [35] [HOMA − IR = fasting serum glucose (mg/dL) X fasting serum insulin (mIU/mL)/405].

Osteocalcin (OC) was measured using an ELISA kit (Immutopics Cat. No 60-1505) [36]. Commercial ELISA kits were used to measure bone alkaline phosphatase (ALP) (CUSABIO TECH-Cat. No. CSB-E11865r) and Tartrate-resistant acid phosphatase (TRAP) (CUSABIO Cat. Nos. CSB-E08491r) [37]. Spectrophotometry utilizing particular diagnostic reagent kits (BioMérieux, France) was used to measure serum calcium [38] and phosphorus [39] levels.

### 2.6. Bone Mineral Density

The right femur was weighed after removal of the soft tissues around it. To obtain the ash, drying the femur bone overnight at 100°C, followed by incineration for 12 hours at 1000°C in muffle apparatus, was carried out. The ash was weighed, solubilized with 6 N HCL, quantitatively transmitted into a volumetric flask, and, then, made up to 100 mL with 6 N HCL [40]. Calcium content in bone ash was detected by an atomic absorption spectrophotometer [41]. Inorganic phosphate content in bone ash was detected by using a spectrophotometer according to Plummer's method [42].

### 2.7. Histological Examination of the Bone

The left femurs were dissected, followed by immediate fixation in neutral buffered formaldehyde for two days. The distal femoral diaphysis was prepared using the decalcification method. Decalcification was carried out for four weeks using the chelating agent ethylene diamine tetraacetic acid in the form of its disodium salt (5.5 g ethylene diamine tetraacetic acid in 90 mL distilled water and 10 mL formaldehyde 37–40%). The decalcifying solution was changed every day [43]. The decalcified samples were dehydrated and processed to get transverse and longitudinal paraffin sections (5 *μ*m thickness). Then, all sections were stained with hematoxylin and eosin stain [44]. The distal femur is one of the most sensitive sites for bone loss four weeks after ovariectomy. Also, the bone loss from it still higher after a more extended period (36 weeks) ~57-64% compared to the spine ~57-64% and cranial bones ~1-3% [45].

### 2.8. Statistical Analysis

Values are presented as a mean ± standard deviation (SD). Statistical analysis was done using one-way analysis of variance (ANOVA) followed by LSD post hoc test for multiple comparisons. Statistical analysis was performed using the Statistical Package for the Social Sciences (SPSS), version 18 (SPSS, Inc., USA). Statistical significance was represented at *P* value ≤ 0.05. Correlations between HOMA-IR and a number of osteoclasts/10 HPF in group III (OVX-control) and group IV (OVX-irisin-treated group) were performed by the Pearson 2-tailed test.

## 3. Results

### 3.1. Biochemical Analysis

Table 1 shows BMI (g/cm2), serum glucose (mg/dL), serum insulin (mIU/mL), HOMA-IR, dry weight (mg/femur), ash weight (mg/femur), and calcium and phosphorus content in bone ash (mg/g bone ash). All values are expressed as means ± SD. The results showed no significant variation for all parameters measured (*P* > 0.05) between the sham-operated and control groups.

The OVX-control group showed a significant rise (*P* < 0.05) in BMI, serum glucose level, serum insulin level, HOMA-IR, with a significant reduction (*P* < 0.05) in dry weight, ash weight, phosphorus, and calcium content in bone ash when compared with the control group, sham-operated group, and OVX-irisin-treated group. In OVX-irisin-treated rats, all parameters are shown to return near their normal levels compared to the control and sham-operated groups.

Table 2 shows the concentration of OC, ALP, TRAP, calcium, and phosphorus in the sera of all studied groups. In group III (OVX-control group), the mean values showed a significant increase for serum OC, ALP, TRAP, calcium, and phosphorus (*P* < 0.05) for all parameters in comparison with that of the control and sham-operated groups.

In group IV (OVX-irisin-treated group), the mean values exhibited a significant decrease for serum OC, ALP, TRAP, calcium, and phosphorus (*P* < 0.05) in comparison with that of group III (OVX-control group). However, all parameters in the serum of group IV (OVX-irisin-treated group) were found to be non-significantly higher (*P* > 0.05) when compared with that of group I and group II (sham-operated group). These results showed that irisin treatment causes the return of bone turnover biomarkers in serum to near its normal levels compared to the control, sham-operated, and OVX-control groups.

### 3.2. Histological Results

**Figure 1:**
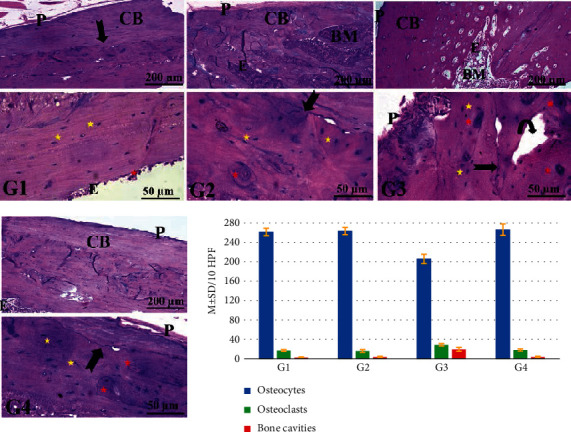
Representative photomicrographs from the longitudinal sections of distal femoral diaphysis revealing the effects of ovariectomy and irisin hormone on the bone structure of rats. The control groups (G I and G II) show the typical bone architecture, outer cortical bone (CB) covered by smooth outer periosteum (P) and lined by smooth endosteum (E). A distinct basophilic cement line (arrow) demarcating between the newly formed bone matrix and the older bone. In contrast, the ovariectomized control rats (G III) reveals loss of typical bone architecture with an irregular periosteal surface (P), an apparent increase in the number of resorbed bone cavities (curved arrow), some osteocytes (yellow star), and numerous osteoclasts (red star), while the ovariectomized rats treated with irisin hormone (G IV) exhibits outer cortical bone (CB) covered by smooth periosteum (P) and inner endosteum (E) containing bone marrow (BM) with numerous osteocytes (yellow star), few osteoclasts (red star), and basophilic cement lines (arrow). In all groups (G I, G II, G III, and G IV), the upper panel is stained with H&E; 100x, bar = 200 *μ*m and the lower panel is stained with H&E; 400x, bar = 50 *μ*m). The histogram (H) of Figure 1 represents the statistical analysis of the number of osteocytes, osteoclasts, and resorbed bone cavities/10 HPF of bone tissue. *P* < 0.05 denotes a significant difference between G IV versus G II and G III.

**Figure 2:**
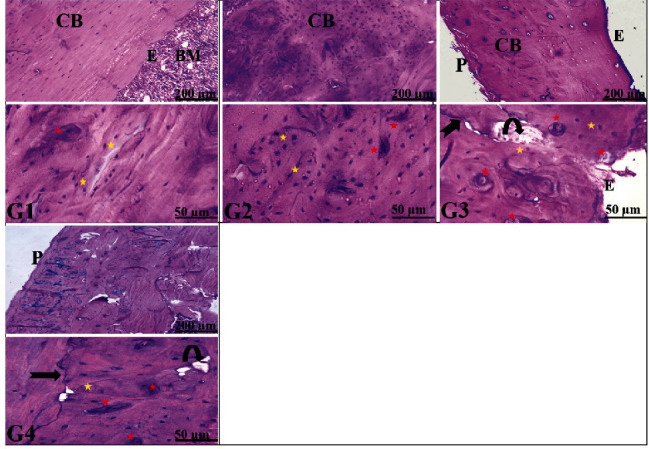
Representative photomicrographs from the transverse sections of distal femoral diaphysis revealing the effects of ovariectomy and irisin hormone on the bone structure of rats. The control groups (G I and G II) show the typical bone architecture, outer cortical bone (CB) covered by smooth outer periosteum (P), lined by smooth endosteum (E), and containing bone marrow (BM). In contrast, the ovariectomized control rats (G III) reveals loss of typical bone architecture with an irregular periosteal surface (P), an apparent increase in the number of resorbed bone cavities (curved arrow), some osteocytes (yellow star), and numerous osteoclasts (red star) indicating resorption of bone in GIII, while the ovariectomized rats treated with irisin hormone group IV (OVX-irisin-treated group)) exhibits outer cortical bone covered by smooth periosteum (P) with numerous osteocytes (yellow star), few osteoclasts (red star), few bone cavities (curved arrow), and basophilic cement lines (arrow), indicating the formation of new bone in G4. In all groups (G I, G II, G III, and G IV), the upper panel is stained with H&E; 100x, bar = 200 *μ*m and the lower panel is stained with H&E; 400x, bar = 50 *μ*m). *P* < 0.05 denotes a significant difference between G IV versus G II and G III.

Histopathological examination of the transverse and longitudinal sections from the distal femoral diaphysis in groups I and II revealed closely similar results. The diaphysis is formed of outer compact cortical bone covered by periosteum (outer fibrous layer and inner osteogenic layer) and inner cancellous bone lined by endosteum (a thin vascular membrane of connective tissue around the bone marrow). The compact cortical bone contains numerous deeply stained small osteocytes and few large acidophilic osteoclasts (multinucleated giant cells residing in Howship's lacunae) with a distinct subperiosteal basophilic cement line (bone deposition demarcating between the newly formed matrix and the older bone) (Figures 1 and 2). In contrast, examination of femoral bone sections from group III revealed loss of typical bone architecture with an apparent increase in the number of osteoclasts and resorbed bone cavities. The cortical bone showed faint basophilic subperiosteal cement lines (which indicates mild deposition of new bone) compared to the control group. In contrast, group IV (ovariectomized rats treated with irisin) exhibited a notable typical bone architecture and distinct lines of subperiosteal bone deposition compared to group III. Besides, group IV revealed smooth periosteal and endosteal surfaces with few subperiosteal resorbed bone cavities, numerous osteocytes, and few osteoclasts (at the areas of prominent new bone deposition).

**Figure 3:**
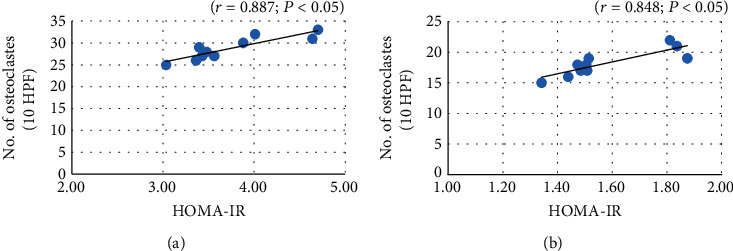
Correlation between HOMA-IR and number of osteoclasts/10 HPF in group III (OVX-control—Figure 3(a)) and group IV (OVX-irisin-treated group—Figure 3(b)).

Statistical analyses of the morphometric measurement in all groups, including the numbers of osteocytes, osteoclasts, and resorbed bone cavities/10 HPF of bone tissue, are represented in the histogram of Figure 1. The number of osteoclasts/10 HPF was correlated positively and significantly with Homeostatic Model Assessment of Insulin Resistance (HOMA-IR) in group III (OVX-control group) (*r* = 0.887; *P* < 0.05) (Figure 3(a)) and group IV (OVX-irisin-treated group) (*r* = 0.848; *P* < 0.05) (Figure 3(b)). These results indicate that irisin may protect against osteoporosis by improving insulin resistance.

## 4. Discussion

Irisin is one of the myokines that has been approved to play several functions which are linked mainly to the recognized advantages of exercise, like reinforcing bone, expanding energy expenditure, and developing cognition [18, 25, 46].

Our findings revealed that bone mineral density decreased in group III (OVX-control group) as the femur's dry and ash weight, phosphorus, and calcium contents in bony ashes were significantly reduced. The effect of ovariectomy on bone mass density (BMD) was approved by Griffith et al. [47], who performed computed tomography (CT) of bone densitometry and imaging of perfusion using magnetic resonance imaging (MRI) at baseline, two, four, and eight weeks following ovariectomy or sham surgery and their results revealed a significant reduction in BMD in concurrent with reduced bone perfusion.

We measured the biochemical markers of bone that are consisted of BALP, TRAP, and OC. Osteoblasts and osteoclasts, respectively, release BALP and TRAP; BALP is a marker for bone mineralization, and TRAP is a marker for bone resorptive action [48, 49]. OC is a serum marker that is reflecting osteoblast activities comprising bone formation and turnover [50]. There was a significant rise in serum levels of OC, BALP, TRAP, calcium, and phosphorus when compared with the control and sham-operated groups. These results were in accordance with the results of Havill et al. [48] and Hassan et al. [51], who revealed that OVX rats showed a significant elevation of serum and bone ALP, OC, and TRACP.

The histological investigation of distal femurs' diaphysis of group III (OVX-control rats) declared a loss of typical bone architecture with an irregular periosteal surface, increasing the number of resorbed cavities, fewer osteocytes, and numerous osteoclasts inside their Howship's lacunae, indicating resorption of bone. These findings are congruous with the results of Mohamed et al. [52], which showed osteoporotic changes in femur diaphysis and metaphysis with a reduction in the mean trabecular bone volume, a widening of the bone marrow space, and a rise in the number of osteoclast cells in OVX groups compared to control and sham-operated control.

Estrogen deficiency causes an acceleration of bone remodeling where osteoclastic bone resorption outpaces osteoblasts' anabolic activity [53] with a high serum level of OC [54]. Moreover, Almeida et al. [55] approved that estrogen deficiency can accelerate the impact of aging on bone by reducing the defensive mechanisms against oxidative stress, causing adverse effects on bone homeostasis. Lack of estrogen triggers the receptor activator of NF-*κ*B ligand (RANKL) production, which is the most potent stimulator of osteoclastogenesis and a suppressor of osteoprotegerin (OPG), a trap receptor for RANKL increasing RANKL bioactivity and bone resorption, with conclusive loss of bone [56]. Group III (OVX control group) showed metabolic dysfunction (increased BMI, insulin level, and insulin resistance). Blüher [17] reported that insulin resistance, a common feature of MS, may be considered a link between MS and chronic degenerative diseases, including osteoporosis. A study conducted by Li et al. [57] showed an alveolar bone loss, osteoclastogenesis, and inflammation with induction of MS (increase in body weight, plasma lipids, insulin, and insulin resistance) in a high-fat diet for 16 weeks in C57Bl/6 mice. Many studies reported a negative correlation between insulin resistance and bone architecture and density [58, 59]. Our results also indicated a positive and significant correlation between insulin resistance and the number of resorbed bone cavities in both groups III and IV. Kim et al. [60] measured the trabecular bone score (TBS), which is a parameter of bone texture. It evaluates pixel grey-level variations in dual-energy X-ray absorptiometry images of the lumbar spine. It is considered an indirect measure of bone quality (decreased TBS reflects the impaired bone quality and microarchitecture). In this study, the TBS was inversely correlated with HOMA-IR in individuals with diabetes. Similarly, Iki et al. [61] reported that TBS was negatively associated with hyperglycemia and insulin resistance.

Weekly irisin treatment in group IV increased the bone mineral density when compared with that of group III (OVX-nontreated group) as the mean values of dry and ash weight of the femur, calcium, and phosphorus contents of bony ashes were found to be significantly increased. The mean values of serum levels of BALP, TRAP, OC, calcium, and phosphorus were significantly decreased. Moreover, BMI and HOMA-IR values have remained close to their average values; this effect may indirectly improve bone health.

The histological analysis of the distal femur diaphysis of group IV (OVX-irisin-treated group) showed typical bone architecture with fewer osteoclasts, bone cavities, and distinct lines of subperiosteal bone deposition compared to group III.

A contemporary study revealed that irisin directly targets osteocytes, which act through the integrin *α*V receptor family. It also studied irisin's effect on sclerostin, which is formed explicitly by osteocytes, produces bone resorption, and regulates bone remodeling. It proved that irisin treatment raised the expression of sclerostin mRNA level in osteocyte cultures in a dose-dependent manner. Besides, they found that injection of recombinant irisin protein daily into mice for six days caused an elevation in sclerostin mRNA level in osteocyte-enriched bone and its level in plasma. These results run against our results and the results of other studies [25, 46], which reported that intermittent injections of irisin with a very low dose (once per week for four weeks) increase cortical bone mineral density and strength in mice suggesting a beneficial effect of irisin in the management of osteoporosis. The contradiction may be explained by an additional possible impact of irisin on other bone cells in the remodeling unit [62]. The intermitted administration may also let irisin positively affect remodeling the same as recorded for parathyroid hormone (PTH), which has an anabolic effect on the skeleton when given intermittently, at least over the initial twelve months of treatment [63, 64]. Moreover, Qiao et al. [65] showed that irisin could enhance osteoblast differentiation through increasing the expression of osteoblastic transcription regulators such as Runt-related transcription factor-2, osterix/sp7, and osteoblast differentiation markers, including alkaline phosphatase, osteocalcin, collagen type 1 alpha-1, and osteopontin. Moreover, it increases ALP activity and calcium deposition in cultured osteoblasts.

In vitro study of Zhang et al. [66] discovered that irisin increased bone trabecular density and cortical thickness in mice by stimulating osteoblasts' differentiation via the Wnt/p-catenin pathway in osteoblastic MC3T3-E1 cells. Also, irisin inhibited osteoclast differentiation by suppressing the RANKL/nuclear factor of activated T cells [67].

Many other studies approved the excellent effect of irisin on bone density, like Allen and Bloomfield [68] and Swift et al. [69]. They agreed that irisin's partial administration prohibits the growth of disuse-induced osteoporosis and muscular atrophy in hindlimb suspended mice. This murine model imitates counter effects on the musculoskeletal system due to long bed rest, physical immobility, and microgravity exposure in humans. Furthermore, many researchers examined the relation between irisin level and BMD in humans. The study of Singhal et al. [70] demonstrated a positive link of irisin with BMD and bone strength in athletes. As in soccer players, they have shown that irisin and BMD are positively correlated at different anatomical sites. Albrecht et al. [3] and Farr et al. [71] stated that circulating irisin levels were lower than average in patients with type 2 diabetes mellitus, which displayed a higher risk of osteoporosis and bone fractures and in subjects who suffered from osteoporotic fractures.

Moreover, many studies demonstrated low serum irisin levels in postmenopausal women with past osteoporotic fractures [72, 73]. Palermo et al. [24] also determined that irisin levels and vertebral fragility fractures are inversely correlated.

## 5. Conclusions

This study's results affirm that intermitted treatment with irisin has a definite role in bone health and suggests that irisin could be a valuable target for the treatment of postmenopausal osteoporosis. Further experimentation will be required to support the possibility of irisin's therapeutic potential in providing numerous bone health benefits.

## Figures and Tables

**Table 1:** BMI, serum glucose, serum insulin, HOMA-IR, dry weight, ash weight, and calcium and phosphorus content in bone ash in all studies groups.

	Group I (negative control)	Group II (sham operated)	Group III (OVX-nontreated)	Group IV (OVX-irisin treated)
BMI (g/cm^2^)	0.51 ± 0.04	0.52 ± 0.04	0.66 ± 0.12	0.53 ± 0.06
		*P* = 0.51^a^	*P* < 0.01^a,b^	*P* = 0.32^a^, *P* = 0.62^b^, P <0.01^c^
Glucose (mg/dL)	78.99 ± 6.96	79.83 ± 5.78	108.63 ± 7.76	80.23 ± 7.93
		*P* = 0.77^a^	*P* < 0.001^a,b^	*P* = 0.71^a^, *P* = 0.90^b^, *P* < 0.001^c^
Insulin (*μ*IU/mL)	7.97 ± 0.66	7.78 ± 0.53	13.94 ± 1.39	7.99 ± 0.77
		*P* = 0.48^a^	*P* < 0.001^a,b^	*P* = 0.96^a^, *P* = 0.49^b^, *P* < 0.001^c^
HOMA-IR	1.55 ± 0.11	1.54 ± 0.18	3.75 ± 0.55	1.58 ± 0.19
		*P* = 0.87^a^	*P* < 0.001^a,b^	*P* = 0.69^a^, *P* = 0.62^b^, *P* < 0.001^c^
Dry wt. (mg/femur)	494.97 ± 13.66	485.47 ± 19.26	390.11 ± 18.59	484.38 ± 15.79
		*P* = 0.22^a^	*P* < 0.001^a,b^	*P* = 0.13^a^, *P* = 0.89^b^, *P* < 0.001^c^
Ash wt. (mg/femur)	291.98 ± 15.71	285.20 ± 19.34	203.49 ± 15.75	281.96 ± 21.22
		*P* = 0.40^a^	*P* < 0.001^a,b^	*P* = 0.25^a^, *P* = 0.73^b^, *P* < 0.001^c^
Bone Ca (mg/g ash)	154.27 ± 5.71	154.97 ± 6.32	119.86 ± 7.66	150.97 ± 8.51
		*P* = 0.61^a^	*P* < 0.001^a,b^	*P* = 0.44^a^, *P* = 0.25^b^, *P* < 0.001^c^
Bone P (mg/g ash)	39.15 ± 5.45	38.89 ± 7.03	28.66 ± 4.42	38.25 ± 5.51
		*P* = 0.93^a^	*P* < 0.001^a,b^	*P* = 0.72^a^, *P* = 0.82^b^, *P* < 0.001^c^

Significance level at *P* < 0.05. ^a^*P* value of significance versus negative control group, ^b^*P* value of significance versus sham-operated group, and ^c^*P* value of significance versus ovariectomized-nontreated group.

**Table 2:** Concentration of OC, ALP, TRAP, calcium, and phosphorus in sera of all studied groups. *P* > 0.05.

	Group I (negative control)	Group II (sham-operated)	Group III (OVX-nontreated)	Group IV (OVX-irisin treated)
OC (ng/mL)	10.65 ± 1.74	10.88 ± 1.42	15.73 ± 1.12	10.76 ± 1.60
		*P* = 0.76^a^	*P* < 0.001^a,b^	*P* = 0.88^a^, *P* = 0.87^b^, *P* < 0.001^c^
BALP (U/L)	183.03 ± 9.72	187.13 ± 8.62	252.91 ± 7.23	192.11 ± 12.12
		*P* = 0.62^a^	*P* < 0.001^a,b^	*P* = 0.17^a^, *P* = 0.31^b^, *P* < 0.001^c^
TRAP (IU/L)	486.25 ± 14.47	492.50 ± 15.39	661.82 ± 8.89	498.37 ± 17.15
		*P* = 0.36^a^	*P* < 0.001^a,b^	*P* = 0.11^a^, *P* = 0.43^b^, *P* < 0.001^c^
Serum level of Ca (mg/dL)	10.23 ± 0.45	10.33 ± 0.56	12.91 ± 0.84	10.36 ± 0.96
		*P* = 0.65^a^	*P* < 0.001^a,b^	*P* = 0.71^a^, *P* = 0.95^b^, *P* < 0.001^c^
Serum level of P (mg/dL)	4.58 ± 0.33	4.51 ± 0.45	6.03 ± 0.56	4.56 ± 0.64
		*P* = 0.72^a^	*P* < 0.001^a,b^	*P* = 0.95^a^, *P* = 0.85^b^, *P* < 0.001^c^

Significance level at *P* < 0.05. ^a^*P* value of significance versus negative control group, ^b^*P* value of significance versus sham-operated group, and ^c^*P* value of significance versus ovariectomized-nontreated group.

## Data Availability

All data generated or analyzed during this study are included in this published article.

## Nomenclature

ANOVA: Analysis of variance
BALP: Bone alkaline phosphatase
BMD: Bone mass density
BMI: Body mass index
CT: Computed tomography
FNDC5: Fibronectin type III domain (five genes)
HOMA-IR: Homeostatic Model Assessment of Insulin Resistance
MRI: Magnetic resonance imaging
MS: Metabolic syndrome
OC: Osteocalcin
OPG: Osteoprotegerin
OVX: Ovariectomized
RANK: Receptor activator of nuclear factor B ligand
TRAP: Tartrate-resistant acid phosphatase
WAT: White adipose tissue.
